# Differences in the relationship between social capital and hypertension in emerging vs. established economies in Sub-Saharan Africa

**DOI:** 10.1186/s12889-022-13471-8

**Published:** 2022-05-24

**Authors:** Vincent Renta, Rebekah J. Walker, Sneha Nagavally, Aprill Z. Dawson, Jennifer A. Campbell, Leonard E. Egede

**Affiliations:** 1 School of Medicine, Medical College of Wisconsin, Milwaukee, WI USA; 2 Department of Medicine, Division of General Internal Medicine, Medical College of Wisconsin, 8701 Watertown Plank Rd., Milwaukee, WI USA; 3 Center for Advancing Population Science (CAPS), Medical College of Wisconsin, Milwaukee, WI USA

**Keywords:** Social capital, Hypertension, Cardiovascular disease, Non-communicable disease, Ghana, South Africa

## Abstract

**Background:**

The global burden of hypertension is growing, particularly in low- and middle-income countries. This study aimed to investigate differences in the relationship between social capital and hypertension between regions in Sub-Saharan Africa (West vs. South Africa) and within regions (rural vs. urban residence within each country).

**Methods:**

Data for 9,800 adults were analyzed from the Study on Global Ageing and Adult Health (SAGE) 2007-2010 for Ghana (West African emerging economy) and South Africa (South African established economy). Outcomes were self-reported and measured hypertension. The primary independent variable was social capital, dichotomized into low vs. medium/high. Interaction terms were tested between social capital and rural/urban residence status for each outcome by country. Linear and logistic regression models were run separately for both countries and each outcome.

**Results:**

Those with low social capital in the emerging economy of Ghana were more likely to have hypertension based on measurement (OR=1.35, 95% CI=1.18,1.55), but the relationship with self-reported hypertension lost significance after adjustment. There was no significant relationship in the relationship between social capital and hypertension in the established economy of South Africa after adjustment. No significant interactions existed by rural/urban residence status in either country.

**Conclusion:**

Low social capital was associated with worse hypertension outcomes, however, the relationship differed between South Africa and Ghana. Further investigation is needed to understand differences between and within countries to guide development of programs targeted at leveraging and promoting social capital as a positive component of overall health.

## Background

Sub-Saharan Africa has seen a shift in its burden of disease from infectious to non-communicable diseases (NCDs), coupled with the largest increase in chronic disease incidence worldwide [1–4]. Hypertension is the leading cause of cardiovascular disease worldwide and is estimated to be responsible for 45% of NCD-related deaths in Sub-Saharan Africa [5, 6]. Higher life expectancy, urbanization, poverty, and changing lifestyle practices are some of the forces altering the burden of disease in Sub-Saharan Africa, due to risk factors for hypertension including comorbidities like obesity and history of stroke, older age, and elevated blood glucose [7–9].

**Fig. 1:**
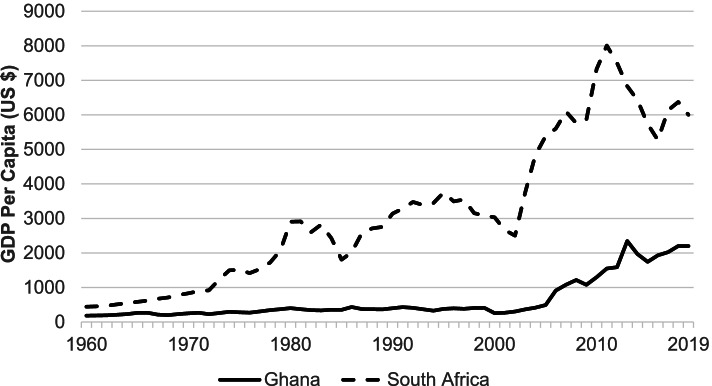
Historical trends in per capita gross domestic product (GDP) for South Africa and Ghana between 1960-2019. Ghana, an emerging economy in West Africa has a projected gross domestic product (GDP) growth rate of 6.1%, while South Africa, an established economy in the South, has a projected GDP growth rate of 1.97%., suggesting more equally distributed growth over the next few years (data source: www.macrotrends.net)

While economic growth in Africa increased in recent years, there is variation in the rate experienced across the continent [10]. Over the past 50 years, the largest expansion has been in the ‘Big 5’ economies of Algeria, Egypt, Morocco, Nigeria, and South Africa, with limited economic growth in other nations [10]. However, recent trends suggest more equally distributed growth over the next few years with East and West Africa being the continent’s fastest growing regions [10]. For example, Ghana, an emerging economy in West Africa has a projected gross domestic product (GDP) growth rate of 6.1%, while South Africa, an established economy in the South, has a projected GDP growth rate of 1.97% [11]. The per capita GDP can be seen in Fig. 1, demonstrating tremendous growth in South Africa, and recent increases in Ghana [12]. Economic growth in Sub-Saharan Africa also varies within countries, with GDP growth concentrated in and reliant on productivity in urban regions [13]. Sub-Saharan Africa is considered one of the fastest urbanizing regions in the world, however, these urban areas are not equipped to mitigate risks associated with this rate of growth, including influences on infrastructure, services, and health within countries [13].

The confluence of growing economies and their influence on increasing incidence of non-communicable diseases has made hypertension a growing concern in Sub-Saharan Africa [14–18]. The rise of hypertension in Sub-Saharan Africa increases economic stress on individuals and health systems because treatment and complications are costly to individuals, and high incidence of disease taxes overburdened health systems that lack sufficient resources [19, 20]. Among Africa’s highest cardiovascular mortality regions, there is an estimated $1.7-3 billion loss in productivity from cardiovascular disease alone as chronic disease can decrease productivity, lead to missed days at work, and have long-term, sometimes costly treatments [21]. Similar to differences in economic growth by rural/urban residence, hypertension is considered uncommon in rural Africa but a major concern in urban Africa [22–25]. As a result, differences by both region and within nations are a concern.

Increasing focus has been placed on social factors that could be used to mitigate the influence of conditions in which people live, work, and play on health outcomes [26]. Social capital, existing when healthy social networks between people, neighborhoods, and organizations offer benefits and resources such as coordination, reciprocity, and cooperation, has been associated with improved health and management of chronic disease [27–30]. It has been suggested that social capital may lower the risk of developing hypertension and its complications, however, little work has been conducted in middle and low income countries [28, 31]. Aspects of social capital, such as trust, personal empowerment, a strong sense of community and safety, and cooperation have all been associated with lower odds of hypertension and/or sedentary behavior, but each factor alone may be less influential than combined [30–35].

Previous studies on social capital have largely focused on the relationship with health behaviors and cognitive health, with less work conducted in chronic conditions, such as hypertension. In addition, studies have not been designed to investigate differences between different regions, or at differences within a nation, such as rural/urban residence status. Given the changing economies and rising incidence of non-communicable diseases in Sub-Saharan Africa, we sought to investigate differences in the relationship between social capital and hypertension between regions in Sub-Saharan Africa (West vs. South Africa) and within regions (rural vs. urban residence within each country).

## Methods

### Data and Sample

We analyzed 9,800 adults (≥ 18 years) from the Study on Global Ageing and Adult Health (SAGE) Wave 1 individual data files [36, 37]. No IRB approval was required as this analysis used publicly available data. SAGE Wave 1 was administered from 2007 to 2010 in China, Ghana, India, Mexico, Russian Federation and South Africa. Based on the objective of this study, we selected two countries in Sub-Saharan Africa that measured social capital and both measured and self-reported hypertension (Ghana and South Africa). South Africa has a longer history of economic growth and higher levels of current urbanization, while Ghana is an emerging economy in the fast growing West African region.

Both Ghana and South Africa SAGE data used a stratified multistage cluster sample design. Strata for South Africa was defined by the nine provinces (Eastern Cape, Free State, Gauteng, Kwa-Zulu Natal, Limpopo, Mpumalanga, North West, Northern Cape and Western Cape), locality (urban or rural), and predominant race group (African/Black, White, Colored, and Indian/Asian) [38, 39]. Strata for Ghana were defined by administrative region (Ashanti, Brong Ahafo, Central, Eastern, Greater Accra, Northern, Upper East, Upper West, Volta and Western) and type of locality (urban/rural) [38, 39]. Households with one or more members aged 50 years or more were available for the household interview, and individuals living in the household who were 50 years or older were eligible for individual interview [38, 39]. Household weights for analysis at the person level were calculated by SAGE and used in the analysis to allow weighting to the population level.

Household and individual participant’s health status and health systems assessment were the two primary components of SAGE survey instruments. Additionally, SAGE assessments included participant’s perceptions on well-being and objective measures of health which included anthropometrics, performance tests and biomarkers. The majority of respondents that SAGE interviewed were 50 years and older while a relatively smaller sample were between ages 18 and 49. In both countries, data was collected face-to face using paper and pencil interviews. The individual response rate in Ghana was 80% while the response rate in South Africa was 77% [36, 37]. The total number of individual respondents from Ghana and South Africa was 5,573 and 4,227, respectively.

### Outcome measures

Using both measured blood pressure and self-reported hypertension, we created four hypertension outcomes. Blood pressure was measured at the time of the survey administration using an automatic inflatable cuff [40, 41]. Participants were seated with their wrist at the level of their heart during collection of blood pressure, which was measured three times with one-minute breaks between each measurement [40, 41].

1. Self-reported Hypertension (yes/no): “Has a healthcare professional/doctor ever told you, you have high blood pressure/hypertension?”
2. Measured Hypertension (yes/no): participants with measured systolic pressure ≥ 140 mmHg and diastolic pressure ≥ 90 mmHg were determined as having hypertension.
3. Systolic Blood Pressure (continuous variable)
4. Diastolic Blood Pressure (continuous variable)

### Independent variable

Our primary independent variable was social capital developed using the following four Wave 1 survey items: “How often in the last 12 months have you … ”

- “ … attended any public meeting in which there was a discussion of local or school affairs?”
- “ … met personally with someone you consider to be a community leader?”
- “ … attended any group, club, society, union or organizational meeting?”
- “ … worked with people in your neighborhood to fix or improve something?”

Each question had response options of ‘never’, ‘once or twice per year’, ‘once or twice per month’, ‘once or twice per week’ and ‘daily’. Each of the above-mentioned variable responses were dichotomized by coding ‘never’ and ‘once or twice per year’ as 0 while ‘once or twice per month’, ‘once or twice per week’, and ‘daily’ as 1. The scores for each question were then summed for each individual creating a continuous variable of 0-4 with higher numbers indicating higher levels of social capital. Following prior use of this variable the score was dichotomized into low social capital (score of 0) and medium/high social capital (score of 1-4) [27].

### Covariates

Sociodemographic variables included age (continuous at time of survey), sex (male, female), income (collected by SAGE and categorized into quintiles within a given country), marital status (dichotomized variable by labeling ‘currently married’ or ‘cohabitating’ as ‘with partner’ vs. ‘never married’, ‘widowed’, or ‘separated/divorced’ as ‘without partner’), education (categorized into less than high school level (no formal education, primary school, and secondary school) vs. high school or greater (completed high school, college/university, or post-graduate degree), area of residence (categorized into rural or urban), work status (categorized into working vs. not working based on response to having worked 2 or more days in the prior week) and comorbidities (including arthritis, stroke, angina, diabetes mellitus, chronic lung disease, asthma, depression).

### Statistical Analysis

Preliminary analysis included descriptive statistics to understand sociodemographic differences between South Africa and Ghana. Based on differences between the two countries, all analyses were stratified by country. First, using Chi-square, ANOVA, and t-tests, univariate comparisons were conducted to understand the difference in hypertension status for each of the hypertension outcomes between 1) rural vs. urban residence, and 2) low vs. medium/high social capital. In order to understand the relationship between social capital and hypertension, a series of regression models were then run. First, two separate unadjusted logistic regression models were used to assess the relationship between social capital and a) self-reported hypertension, and b) measured hypertension. Second, two unadjusted linear regression models were run between social capital and a) systolic blood pressure, and b) diastolic blood pressure. Third, interaction terms were tested for each of the unadjusted models between social capital and rural/urban residence on each of the 4 hypertension outcomes. All interaction terms were not significant, so models were not stratified by area of residence. Each model was then adjusted for age, gender, income, marital status, education, area of residence, work status, and comorbidities. Analysis was performed using R version-4.0.0 [42] and significance was based on *p*<0.05.

## Results

Sample characteristics for each country, South Africa (*n*=4,227) and Ghana (*N*=5,573) are presented in Table 1 stratified by rural vs. urban residence, and in Table 2 stratified by low vs. medium/high social capital. Among participants from South Africa, 49.8% reported low social capital while 35.1% from Ghana reported low social capital. In South Africa 66.6% of the participants lived in urban regions while in Ghana 40.9% lived in urban regions. All demographic characteristics between South Africa and Ghana were significantly different except age.

**Table 1:** Sample characteristics for South Africa (*n*=4,227) and Ghana (*n*=5,573) stratified by rural/urban status

	South Africa			Ghana	
	Rural	Urban		Rural	Urban
	*N*=1,411	*N*=2,810		*N*=3,290	*N*=2,281
**Social capital**^a^			**Social capital**^a^		
Low	39.74%	54.79%	Low	31.42%	40.37%
Medium/High	60.26%	45.21%	Medium/High	68.58%	59.63%
**Age**^a^	60.9 (13.1)	59.9 (11.9)	**Age**^a^	60.5 (14.1)	59.7 (13.9)
**Gender**			**Gender**^a^		
Male	44.15%	41.74%	Male	54.17%	45.46%
Female	55.85%	58.26%	Female	45.83%	54.54%
**Marital Status**			**Marital Status**^a^		
Single	47.09%	46.86%	Single	35.64%	43.86%
w/Partner	52.91%	53.14%	w/Partner	64.36%	56.14%
**Education**^a^			**Education**^a^		
No formal education	70.83%	45.23%	No formal education	89.64%	71.36%
<High School	26.36%	45.72%	<High School	5.50%	14.77%
>=High School	2.81%	9.06%	>=High School	4.86%	13.87%
**Work Status**			**Work Status**^a^		
Working	32.44%	32.20%	Working	78.43%	65.67%
Not-Working	67.56%	67.80%	Not-Working	21.57%	34.33%
**Comorbidities**			**Comorbidities**		
Arthritis^a^	224 (16.8%)	648 (24.1%)	Arthritis	339 (11.3%)	241 (11.6%)
Stroke	39 (2.9%)	103 (3.8%)	Stroke^a^	47 (1.6%)	73 (3.5%)
Angina^a^	60 (4.5%)	169 (6.3%)	Angina	85 (2.8%)	64 (3.1%)
Diabetes^a^	64 (4.8%)	305 (11.3%)	Diabetes^a^	58 (1.9%)	119 (5.7%)
Lung Disease	31 (2.3%)	60 (2.2%)	Lung Disease ^a^	10 (0.3%)	18 (0.9%)
Asthma^a^	44 (3.3%)	130 (4.8%)	Asthma	95 (3.2%)	78 (3.7%)
Depression^a^	20 (1.5%)	108 (4.0%)	Depression	33 (1.1%)	36 (1.7%)

^a^ indicates *p*<0.05 for comparison between rural and urban residence within country using t-test, ANOVA or chi square tests for comparisons.

**Table 2:** Sample characteristics for South Africa (*n*=4,227) and Ghana (*n*=5,573) stratified by social capital

	South Africa			Ghana	
	Low social capital *N*=1,989	Med/High social capital *N*=2,006		Low social capital *N*=1,784	Med/High social capital *N*=3,301
**Age**^a^	61.4 (12.7)	59.3 (11.8)	**Age**^a^	62.9 (14.6)	58.7 (13.6)
**Gender**^a^			**Gender**^a^		
Male	39.36%	45.51%	Male	44.90%	56.95%
Female	60.64%	54.49%	Female	55.10%	43.05%
**Marital status**^a^			**Marital Status**^a^		
Single	51.20%	43.18%	Single	48.68%	35.38%
w/Partner	48.80%	56.82%	w/Partner	51.32%	64.62%
**Education**			**Education**		
No formal education	55.85%	53.08%	No formal education	83.37%	81.73%
<High School	37.60%	40.06%	<High School	8.14%	9.79%
>=High School	6.55%	6.86%	>=High School	8.49%	8.48%
**Area of residence**^a^			**Area of Residence**^a^		
Rural	26.54%	39.89%	Rural	52.91%	62.41%
Urban	73.46%	60.11%	Urban	47.09%	37.59%
**Work status**^a^			**Work Status**^a^		
Working	24.36%	39.24%	Working	59.72%	80.21%
Not-working	75.64%	60.76%	Not-Working	40.28%	19.79%
**Comorbidities**			**Comorbidities**		
Arthritis^a^	548 (27.6%)	324 (16.2%)	Arthritis	192 (10.8%)	388 (11.8%)
Stroke^a^	99 (4.9%)	45 (2.2%)	Stroke^a^	62 (3.5%)	57 (1.7%)
Angina^a^	143 (7.2%)	84 (4.2%)	Angina^a^	72 (4.0%)	77 (2.3%)
Diabetes^a^	216 (10.9%)	154 (7.7%)	Diabetes^a^	75 (4.2%)	101 (3.1%)
Lung disease^a^	56 (2.8%)	33 (1.7%)	Lung Disease	12 (0.7%)	16 (0.5%)
Asthma^a^	107 (5.4%)	66 (3.3%)	Asthma	70 (3.9%)	103 (3.1%)
Depression	67 (3.4%)	59 (2.9%)	Depression	19 (1.1%)	50 (1.5%)

^a^ indicates *p*<0.05 for comparison between low and medium/high social capital within country using t-test, ANOVA or chi square tests for comparisons.

Table 3 describes the hypertension outcomes by level of social capital in South Africa and Ghana, while Table 4 describes the hypertension outcomes by rural/urban status. There was a significant difference between those with medium/high vs. low social capital for self-reported hypertension in South Africa (25.4% vs. 31.7%, *p*<0.001) and Ghana (10.6% vs. 14.7%, *p*<0.001). The mean systolic blood pressure was significantly different between medium/high (M=143.0, SD=26.7) and low (M=144.9, SD=27.1) social capital groups in South Africa (*p*=0.02) and between medium/high (M=132.4, SD=24.75) and low (M=137.7, SD=26.7) social capital groups in Ghana (*p*<0.001). In Ghana, among those with medium/high social capital, the mean diastolic blood pressure was significantly lower (M=87.8, SD=16.7), compared to those with low social capital (M=90.4, SD=17.5) (*p*<0.001). Additionally, in Ghana, there was a significant difference by social capital level for measured hypertension (medium/high: 27.04% vs. low: 35.26%, *p*<0.001).

**Table 3:** Hypertension outcomes by level of social capital in South Africa and Ghana

	South Africa		*p* value	Ghana		*p* value
	Social Capital			Social Capital		
	Low (*n*=1,989)	Med/High (*n*=2,006)		Low (*n*=1,784)	Med/High (*n*=3,301)	
**Hypertension (self-reported)**			**< 0.001**			**< 0.001**
No	68.34%	74.60%		85.26%	89.36%	
Yes	31.66%	25.41%		14.74%	10.64%	
**Hypertension (≥140/90)**			0.06			**< 0.001**
No	53.59%	56.57%		64.74%	72.96%	
Yes	46.41%	43.43%		35.26%	27.04%	
**Systolic Blood Pressure** *Mean (SD)*	144.9 (27.1)	143.0 (26.7)	**0.024**	137.7 (26.7)	132.4 (24.5)	**< 0.001**
**Diastolic Blood Pressure** *Mean (SD)*	95.0 (18.1)	95.2 (18.4)	0.699	90.4 (17.5)	87.8 (16.7)	**< 0.001**

Bold indicates significance at the *p*<0.05 level using t-test and chi square tests for comparisons

**Table 4:** Hypertension outcomes by rural/urban status in South Africa and Ghana

	South Africa		*p* value	Ghana		*p* value
	Rural *N*=1,411	Urban *N*=2,810		Rural *N*=3,290	Urban *N*=2,281	
**Hypertension (self-reported)**			**< 0.001**			**< 0.001**
No	78.10%	68.30%		93.50%	79.70%	
Yes	21.90%	31.70%		6.50%	20.30%	
**Hypertension (≥140/90)**			0.598			**< 0.001**
No	54.70%	55.50%		72.30%	66.80%	
Yes	45.30%	44.50%		27.70%	33.20%	
**Systolic Blood Pressure**	143.6 (27.3)	144.2 (26.7)	0.483	132.1 (25.3)	137.4 (25.4)	**< 0.001**
*Mean (SD)*						
**Diastolic Blood Pressure**	95.8 (18.6)	94.7 (18.0)	0.063	87.5 (17.3)	90.3 (16.5)	**< 0.001**
*Mean (SD)*						

Bold indicates significance at the *p*<0.05 level using t-test and chi square tests for comparisons.

Table 5 shows the unadjusted and adjusted model results for the relationship between social capital and each hypertension outcome in South Africa and Ghana. Participants in South Africa with low social capital were more likely to self-report having hypertension when compared to participants with medium/high social capital (OR=1.36, 95%CI=1.18, 1.56). Additionally, participants in South Africa with low social capital had 1.93 mmHg higher systolic blood pressure when compared to the medium/high social capital group (95%CI=0.25, 3.60). In Ghana participants with low social capital were more likely to self-report having hypertension (OR=1.45, 95%CI=1.22, 1.72), more likely to have measured hypertension (OR=1.47,95%CI=1.30, 1.67), have higher mean systolic blood pressure (5.36, 95% CI=3.90, 6.83), and higher diastolic blood pressure (2.60, 95% CI=1.62, 3.59) compared to the medium/high social capital group. After adjustment, participants with low social capital in Ghana were more likely to have measured hypertension (OR=1.35, 95%CI=1.18, 1.55), higher systolic blood pressure (3.25, 95% CI=1.71, 4.79), and higher diastolic blood pressure (2.37, 95% CI=1.32, 3.41) compared to the medium/high social capital group. All relationships in South Africa lost significance after adjustment.

**Table 5:** Unadjusted and adjusted relationship between social capital and hypertension by country

	South Africa		Ghana	
	Unadjusted	Adjusted	Unadjusted	Adjusted
**Odds Ratio for Hypertension (self-reported)**				
High Social Capital	Ref	Ref	Ref	Ref
Low Social Capital	**1.36 (1.18, 1.56)**	0.89 (0.72, 1.09)	**1.45 (1.22, 1.72)**	1.13 (0.93, 1.39)
**Odds Ratio for Hypertension (≥140/90)**				
High Social Capital	Ref	Ref	Ref	Ref
Low Social Capital	1.13 (0.99, 1.28)	1.14 (0.96, 1.35)	**1.47 (1.30, 1.67)**	**1.35 (1.18, 1.55)**
**Beta Coefficient for Systolic Blood Pressure** (mean difference in mmHg)	**1.93 (0.25, 3.60)**	2.32 (0.04, 4.60)	**5.36 (3.90, 6.83)**	**3.25 (1.71, 4.79)**
**Beta Coefficient for Diastolic Blood Pressure** (mean difference in mmHg)	-.23 (-1.36, 0.91)	-.02 (-1.61, 1.56)	**2.60 (1.62, 3.59)**	**2.37 (1.32, 3.41)**

Adjusted models include age, gender, income quintile, marital status, education, area of residence, work status, comorbidities (arthritis, stroke, angina, diabetes, chronic lung disease, asthma, depression)

Bold indicates significance at the *p*<0.05 level

Logistic regression used to report OR of Hypertension and linear regression used to report coefficient of blood pressure.

## Discussion

Using the Study on Global Ageing and Adult Health (SAGE) dataset, this study found that the relationship between social capital and hypertension differed between South Africa (an established economy in the south of Africa) and Ghana (an emerging economy in the west of Africa). Individuals in Ghana reported higher social capital, and the relationship between social capital and hypertension was stronger in Ghana, with low social capital associated with higher levels of hypertension. After adjustment individuals with low social capital had 35% higher odds of having hypertension as measured by their blood pressure (i.e., ≥ 140/90), and had 3.3 mmHg higher systolic blood pressure and 2.4 mmHg higher diastolic blood pressure after adjustment. The relationship between social capital and self-reported hypertension lost significance after adjustment. Finally, there was no differential relationship in either country by rural vs. urban status for the relationship between social capital and hypertension.

This study adds to the literature by providing information on differences in the relationship between social capital and hypertension. As countries develop, they may lose social capital, and thus an aspect of the protective nature of social connectedness in the nation. There was no indication, however, that this relationship was based on area of residence, and thus urbanization in itself may not be the reason for this difference. Instead, based on these findings growth in GDP over time and overall economic development in a country may be more likely to explain if social capital is an ideal focus for intervention. Prior studies on social capital and health have primarily targeted populations of higher income nations and were largely focused on relationships between social capital and health behaviors and/or aspects of cognitive health [28, 32].

Based on these findings, social capital may be an area to incorporate into programs targeting chronic disease prevention, however, they should be adapted for the location and culture in which they are implemented. Despite the scarcity of social capital intervention studies, there are some which show social capital interventions that promote community engagement and social participation are associated with better physical health [43–45]. In addition, though prior research on depression suggested differences may exist in the relationship with social capital by area of residence [46, 47], this study found that there was no differential relationship between hypertension and social capital by rural/urban status. Detailed investigation into how social capital is built and how that influences health in a variety of situations will be necessary to understand this complex relationship.

This study also highlights the value of having information on both measured and self-reported hypertension as there were differences for measured vs. self-reported hypertension when investigating the relationship with social capital. Future surveys and studies conducted in Sub-Saharan Africa should include measured blood pressure in addition to self-reported hypertension for the same individual to allow comparison between the two and investigation into undiagnosed hypertension.

There are limitations in this study worth noting. First, as the mean age was 60 in both countries, this analysis is representative of an older population, and the relationship may differ in younger populations. Second, the analysis used definitions of social capital that were developed by the World Health Organization, which were available in the dataset. Other measures of this concept may result in different findings given the importance of questions included in a scale on how participants respond. As this scale has been validated in international populations, for the purpose of this analysis it is a valid and reliable measure of social capital, but other measures should be investigated in the future. Third, the study was conducted on data from two countries in Sub-Saharan Africa and may not be representative of other countries. Fourth, the concept of urban and rural environments within Sub-Saharan Africa can differ by country and region, and the understanding of specific concepts, such as days spent working, may differ between urban and rural environments in Sub-Saharan Africa. As such, future work should capture more nuanced aspects of the rural and urban environment to better understand possible differences and similarities in relationships. Finally, the data is cross-sectional in nature and thus causality cannot be inferred from the results.

In summary, through analysis of data in two countries in Sub-Saharan Africa, we found that low social capital is associated with worse hypertension outcomes in the emerging economy of Ghana, but not in the established economy of South Africa. In addition, we found there was no differential relationship by area of residence, suggesting social capital may be a protective social factor in both urban and rural environments. Further investigation is needed on the mechanisms that exist between social capital and hypertension, as well as other contextual factors that may influence the relationship.

## Data Availability

The dataset generated and analyzed during the current study is publicly available and can be accessed at https://apps.who.int/healthinfo/systems/surveydata/index.php/catalog/6/related-materials and https://apps.who.int/healthinfo/systems/surveydata/index.php/catalog/5/related-materials.
